# Clinical Characteristics, Prognostic Factors and Therapeutic Strategies in Gastric Cancer Patients With Bone Metastasis: A Retrospective Analysis

**DOI:** 10.1002/cam4.70781

**Published:** 2025-03-19

**Authors:** Shiji Ren, Yutao Wei, Wenqi Liu, Yipeng Zhang, Yue Wang, Ju Yang, Baorui Liu, Tao Shi, Jia Wei

**Affiliations:** ^1^ Department of Oncology Nanjing Drum Tower Hospital, Affiliated Hospital of Medical School, Nanjing University Nanjing China; ^2^ Department of Oncology Nanjing Drum Tower Hospital Clinical College of Nanjing University of Chinese Medicine Nanjing China; ^3^ Department of Oncology Nanjing Drum Tower Hospital Clinical College of Nanjing Medical University Nanjing China; ^4^ Nanjing Medical Key Laboratory of Oncology Nanjing China

**Keywords:** bone metastasis, gastric cancer, immunotherapy, poorly cohesive, prognosis

## Abstract

**Background:**

Bone metastases are highly refractory and are associated with extremely poor survival. Despite the increasing incidence of bone metastasis in gastric cancer (GC), comprehensive analyses regarding the clinicopathological features, prognosis, and treatment of bone‐metastatic GC remain limited.

**Methods:**

We obtained data from 120 bone‐metastatic GC patients from Nanjing Drum Tower Hospital and 36,139 GC patients from the SEER database. Chi‐square and Mann–Whitney *U*‐tests evaluated clinicopathological features, while Cox models identified prognostic factors. Kaplan–Meier curves and forest plots assessed the effects of different treatment strategies on overall survival after bone metastasis (OS‐BM).

**Results:**

Among 120 bone‐metastatic GC patients, 55 (45.83%) were diagnosed with poorly cohesive gastric carcinoma (PCC). The higher incidence of bone metastasis was also observed in SRCC patients from the SEER database (*p* < 0.0001). PCC patients exhibited distinct pathological features compared to non‐PCC patients, including lower PD‐L1 (*p* = 0.042) and E‐cadherin expression (*p* = 0.049). Multivariate analysis identified various negative prognostic factors such as metachronous bone metastasis (*p* < 0.001, HR = 2.35, 95% CI:1.47–3.74) and CA125 expression (*p* = 0.036, HR = 1.60, 95% CI:1.03–2.48), whereas immunotherapy was a positive prognostic factor (*p* < 0.001, HR = 0.44, 95% CI:0.29–0.66). Subgroup analysis also showed improved survival among different populations of bone‐metastatic GC patients receiving immunotherapy. Moreover, combinational therapies including immunotherapy and other treatments (anti‐angiogenic therapy and/or local radiotherapy) further improved patient OS‐BM.

**Conclusion:**

Our results suggest bone‐metastatic GC patients exhibit distinct clinicopathological features, with a high incidence of bone metastasis in PCC. Immunotherapy‐based combination therapies offer improved survival benefits, thus supporting the application of immunotherapy in GC patients at high risk of bone metastasis.

## 1 Introduction

Gastric cancer (GC) is one of the most prevalent malignancies of the digestive system, ranking fifth in both incidence and mortality rates among cancers worldwide [1]. Bone is a common metastatic site for various solid tumors, and the incidence of bone metastasis is rising in gastrointestinal cancers, including GC [2, 3, 4, 5]. Bone metastasis presents significant challenges due to its asymptomatic onset, lack of early diagnostic markers, and poorly defined prognostic factors, making effective management difficult [6, 7, 8]. Thus, it is necessary to further explore the clinicopathological characteristics and treatments for GC patients with bone metastasis.

Poorly cohesive gastric carcinoma (PCC) is a distinct histological subtype of GC, characterized by isolated or small clusters of tumor cells that lack glandular formation and exhibit a poorly cohesive growth pattern [9, 10]. PCC patients exhibit unique epidemiological and clinical characteristics, including a younger age of onset and a higher propensity for metastasis [11]. Single‐cell analysis has revealed that the PCC subtype exhibits lower cell adhesion, higher immune evasion capabilities, and an immunosuppressive microenvironment, which may be closely linked to its aggressiveness [12, 13]. Despite curative surgery, PCC patients often face a high risk of distant metastasis and may develop resistance to chemotherapy and immunotherapy [14, 15, 16]. However, the clinicopathological features, prognostic factors, and potential biomarkers of PCC patients, especially those with bone metastasis, remain poorly understood.

Cancer immunotherapies such as immune checkpoint blockade (ICB) have achieved remarkable breakthroughs in a number of primary tumors [17, 18, 19, 20, 21]. However, both preclinical and large population‐based clinical studies on cancers such as lung, breast, and prostate cancer indicate that bone metastases have extremely poor responses to anti‐programmed cell death protein‐1 (PD‐1) or cytotoxic T‐lymphocyte antigen‐4 (CTLA‐4) therapy [22, 23, 24, 25, 26]. For the treatment of advanced GC, immunotherapy has achieved satisfactory results, breaking through the long‐standing treatment bottleneck of short survival with traditional chemotherapy. The phase III CheckMate 649 trial [27] showed that nivolumab combined with chemotherapy increased mOS by 3.3 months and decreased the risk of death by 29% compared to chemotherapy alone in the PD‐L1 CPS ≥ 5 patients. Subsequently, ORIENT‐16 [28], KEYNOTE‐859 [29], RATIONALE‐305 [30], and GEMSTONE‐303 [31] showed that combination chemotherapy with immunotherapy significantly increased mOS for GC, especially in patients with high PD‐L1 expression. However, these clinical trials mostly focused on advanced GC with lymph node or visceral metastasis, while prospective clinical trials evaluating the efficacy of immunotherapy in patients with bone metastasis are lacking. Meanwhile, retrospective studies on GC with bone metastasis have mainly concentrated on palliative surgery or chemotherapy, with limited analysis of the potential benefits of immunotherapy in this specific population.

In this study, we analyzed the clinicopathological features of 120 GC patients with bone metastasis from Nanjing Drum Tower Hospital Cancer Center, with a particular focus on the PCC subtype. We aimed to identify the prognostic factors that influence overall survival after bone metastasis (OS‐BM) and constructed a nomogram for GC patients with a high risk of bone metastasis. Moreover, we assessed the OS‐BM of bone‐metastatic GC patients receiving various treatment strategies, including immunotherapy, thereby providing new findings for optimizing the treatment options for advanced GC patients with bone metastasis.

## 2 Methods

### 2.1 Study Population and Data Collection

**FIGURE 1:**
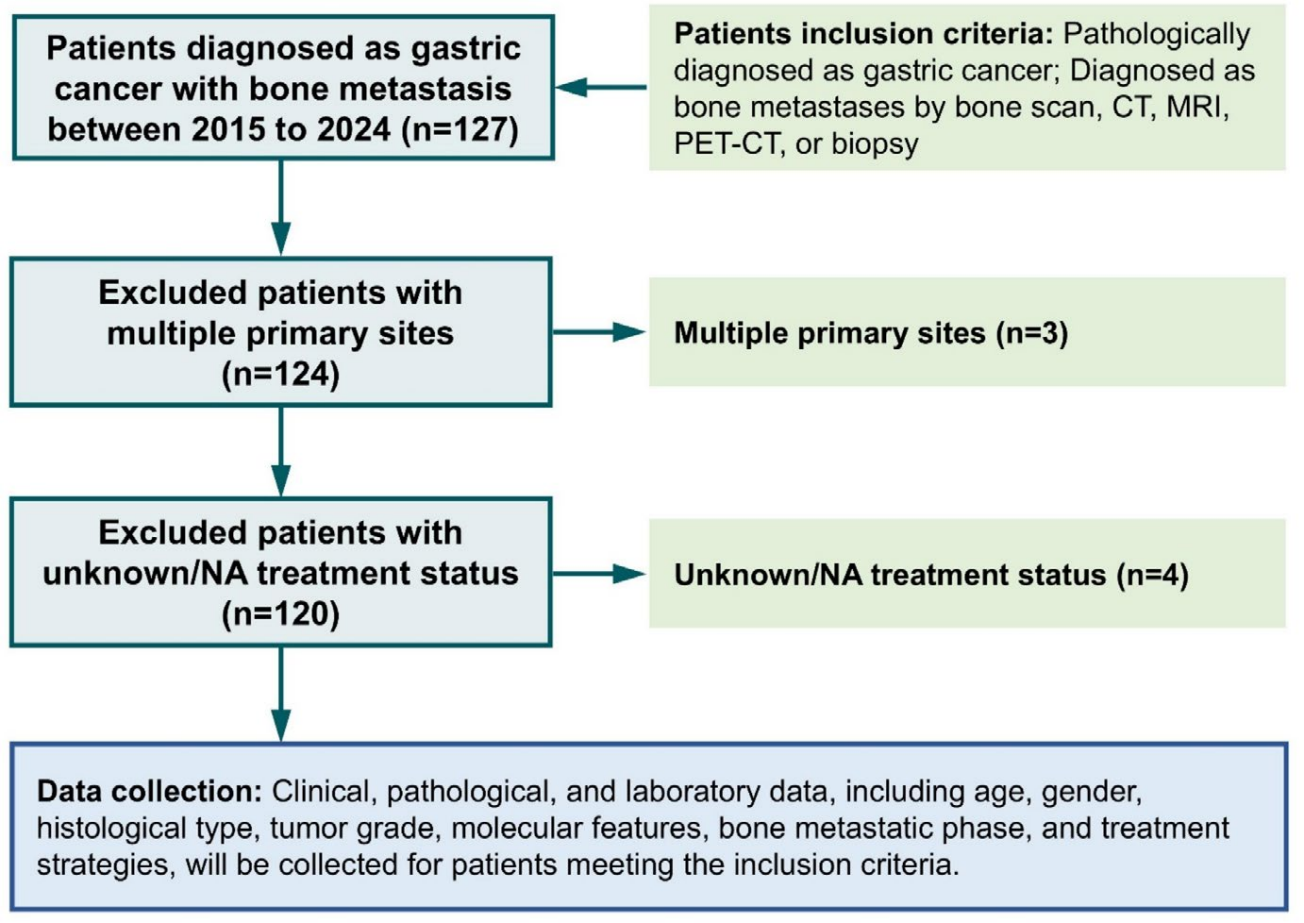
Flow chart of the inclusion and exclusion process of gastric cancer patients with bone metastasis from Nanjing Drum Tower Hospital Cancer Center.

We conducted a retrospective study of gastric cancer (GC) patients with bone metastasis at Drum Tower Hospital between 2015 and 2024. Patients were selected based on the following inclusion criteria: (a) a pathologically confirmed diagnosis of gastric cancer; (b) bone metastasis verified by radiological or histological methods, including bone scan, CT, MRI, PET‐CT, or biopsy (Figure 1). Exclusion criteria included patients with multiple primary cancer sites and those with unknown treatment status.

For all eligible patients, clinical and pathological data were collected, including age, gender, histological type, tumor grade, bone metastatic phase, treatment strategies, among others. The bone metastatic phase was categorized as synchronous or metachronous. Synchronous bone metastasis refers to bone metastasis detected during the initial diagnosis of gastric cancer, whereas metachronous bone metastasis was identified during follow‐up or subsequent treatments after the initial gastric cancer diagnosis. Laboratory data at the time of bone metastasis diagnosis were also recorded, encompassing serum levels of alkaline phosphatase, lactate dehydrogenase, calcium, albumin, tumor markers, among others.

### 2.2 Data Extraction From the SEER Database

The Surveillance, Epidemiology, and End Results (SEER) database, which has collected detailed patient information across various regions of the United States since 1973, was used to obtain supplementary data for this study. The SEER database includes information on age, gender, race, year of diagnosis, histological type, TNM staging, and distant organ metastasis. Since 2010, SEER has provided specific information on bone metastases in gastric cancer patients. For this study, we extracted pathologically diagnosed cases of gastric cancer from 2010 to 2021 using SEER*Stat software. The selection criteria included the following: (a) histological confirmation of gastric adenocarcinoma diagnosis, (b) exclusion of patients with unknown bone metastatic status, and (c) exclusion of patients with multiple primary tumor sites. A total of 47,724 gastric cancer patients were identified, among whom 2736 patients were diagnosed with bone metastasis (Figure S1).

### 2.3 Survival Analysis

Survival data were obtained from medical records and follow‐up information. The primary endpoint of the study was overall survival after bone metastasis (OS‐BM), defined as the time from the initial detection of bone metastasis until death from any cause or until the last follow‐up. In addition to the primary endpoint, the interval from the initial diagnosis of gastric cancer to the onset of bone metastasis was defined as bone metastasis‐free survival (BMFS).

### 2.4 Construction and Validation of Prognostic Prediction Models

We developed a nomogram to predict OS‐BM in GC patients with bone metastasis. Variables for constructing the prediction model were screened by univariate and multivariate Cox regression analysis. Points were assigned for each prognostic factor based on the estimated coefficients from the Cox model. The total points for each patient were calculated by summing the scores across the variables. The receiver operating characteristic (ROC) curves were constructed to evaluate the predictive accuracy of the nomogram. The area under the curve (AUC) with 95% confidence intervals (CI) was calculated to assess the discriminatory power of the nomogram at each time point. An AUC value closer to one indicates a better discriminative ability of the model. It is important to note that the cutoff values for laboratory data (such as CEA, CA125, WBC, and LDH) were determined based on established clinical thresholds for abnormal values.

### 2.5 Construction and Visualization of Cox Proportional Hazards Regression Models

Cox proportional hazards regression models were used to estimate the hazard ratios (HR) and their 95% confidence intervals (CI) for various clinicopathological features associated with OS‐BM. The HR and CI for each variable were calculated for both the immunotherapy and non‐immunotherapy groups. The forest plot was constructed to visually display the effect of each variable on survival. Each covariate's HR and 95% CI were represented graphically by horizontal lines, with a square marker indicating the point estimate of the HR. The length of the line represented the 95% CI, and a vertical dashed line was drawn at HR = 1, representing no effect on survival.

### 2.6 Statistical Analysis

Data processing and analysis were performed using R 4.2.0 (http://www.R‐project.org/), along with Zstats v1.0 (www.zstats.net). Chi‐square test (χ^2^ test) and Mann–Whitney *U*‐test were performed to compare the distribution between groups based on responses for categorical variables and continuous variables, respectively. The Kaplan–Meier method was used to perform survival analysis, with *p*‐values compared by the log‐rank test. Also, to analyze the prognostic factors of GC patients with bone metastasis, we employed univariate and multivariate Cox regression models to estimate HR and 95% CI. The forest plot was used to compare the impact of immunotherapy on patients. All statistical tests were performed two‐sided, and *p* < 0.05 were considered to be statistically significant.

## 3 Results

### 3.1 Baseline Characteristics of GC Patients With Bone Metastasis

After patient inclusion (*n* = 127) and exclusion (*n* = 7), a total of 120 GC patients who were diagnosed as stage IV with bone metastases from 2015 to 2024 in Nanjing Drum Tower Hospital were included for the following analysis in this study (Figure 1). The age of the patients ranged from 25 to 81 years, with a median age of 61. Of these, 71 were males (59.17%) and 49 were females (40.83%). Regarding histological subtypes, poorly cohesive gastric carcinoma (PCC, *n* = 55), which the World Health Organization (WHO classification code 8490/3) defines as a subtype characterized by isolated or small clusters of tumor cells that lack glandular formation and show poorly cohesive growth, includes signet ring cell carcinoma (SRCC) and accounted for nearly half of all bone metastatic GC patients. In terms of metastatic sites, 16 patients (13.33%) had bone‐only metastasis, while the majority (104 patients, 86.67%) had metastases at multiple locations. Synchronous bone metastasis was observed in 36 patients (30.00%), while 84 patients (70.00%) had metachronous bone metastasis. All patients received chemotherapy and bone‐targeted agents, including bisphosphonates or denosumab. Additionally, 59 patients (49.17%) underwent immunotherapy (with anti‐PD1/PDL1 agents), 58 patients (48.33%) received anti‐angiogenic therapy with VEGF or tyrosine kinase inhibitors, and 26 patients (21.67%) received local radiotherapy for bone metastases (Table 1). Other details of patients' characteristics are presented in Table 1.

**TABLE 1:** Clinicopathological characteristics of gastric cancer patients with bone metastasis (*n* = 120).

Characteristics	Bone metastasis (*n* = 120)
Age (years), *n* (%)	
< 60	56 (46.67)
≥ 60	64 (53.33)
Gender, *n* (%)	
Male	71 (59.17)
Female	49 (40.83)
Lauren classification^a^, *n* (%)	
Intestinal type	10 (20.00)
Diffuse type	27 (54.00)
Mixture	13 (26.00)
Histological type, *n* (%)	
Poorly cohesive gastric carcinoma	55 (45.83)
Non‐poorly cohesive gastric carcinoma	65 (54.17)
Metastatic site, *n* (%)	
Bone‐only metastasis	16 (13.33)
Multiple metastases	104 (86.67)
Bone metastatic phase, *n* (%)	
Synchronous	36 (30.00)
Metachronous	84 (70.00)
Her‐2^a^, *n* (%)	
‐, +, ++	83 (69.17)
+++, ++ with FISH+	13 (10.83)
VEGFR2^a^, *n* (%)	
Negative	17 (35.42)
Positive	31 (64.58)
E‐cadherin^a^, *n* (%)	
Negative	6 (12.50)
Positive	42 (87.50)
MMR status^a^, *n* (%)	
pMMR	68 (94.44)
dMMR	4 (5.56)
PD‐L1^a^, *n* (%)	
CPS < 1	34 (45.33)
1 ≤ CPS < 10	23 (30.67)
CPS ≥ 10	18 (24.00)
Treatment strategy, *n* (%)	
Chemotherapy	120 (100)
Bone‐targeted agents^b^	120 (100)
Immunotherapy	59 (49.17)
Anti‐angiogenic therapy	58 (48.33)
Local radiotherapy for bone metastases	26 (21.67)

Abbreviation: CPS: combined positive score.

^a^ Indicates missing data for certain patients in the respective categories. These missing entries are due to incomplete records.

^b^ Bone‐targeted agents include bisphosphonates and denosumab.

At the time of bone metastasis diagnosis, various laboratory parameters were recorded. The median (M), first quartile (Q1), and third quartile (Q3) values for key laboratory data are presented in Table S1. Notably, the median carcinoembryonic antigen (CEA) level was 12.36 ng/mL (Q1: 2.30, Q3: 78.17), and the median carbohydrate antigen 19–9 (CA19‐9) level was 37.78 U/L (Q1: 10.08, Q3: 514.75). The median white blood cell (WBC) count was 5.70 × 10^9^/L (Q1: 4.30, Q3: 7.12), and the median neutrophil count was 3.90 × 10^9^/L (Q1: 2.98, Q3: 5.60). Lactate dehydrogenase (LDH) levels were elevated, with a median of 231.00 U/L (Q1: 184.00, Q3: 322.25), while alkaline phosphatase (ALP) levels were also elevated, with a median of 159.65 U/L (Q1: 92.00, Q3: 297.60). C‐reactive protein (CRP) levels showed considerable variability, with a median of 7.85 mg/L (Q1: 3.40, Q3: 26.83).

### 3.2 PCC Patients Have a Higher Incidence of Bone Metastasis Compared to Non‐PCC Patients

To further characterize the clinicopathological features of GC patients with bone metastasis, particularly those with poorly cohesive types, we analyzed data from the SEER database spanning 2010 to 2021. The analysis included 36,139 eligible GC patients, of whom 2144 were diagnosed with bone metastasis (Figure S1). Although the SEER database does not specifically include data on PCC as a distinct pathological type, the findings related to signet ring cell carcinoma (SRCC), a recognized subtype of PCC characterized by more than 90% poorly cohesive cells with signet ring cell morphology, can offer insights into the clinical features of PCC. Given their shared characteristics of low adhesion, the results observed in SRCC patients may reflect underlying patterns relevant to PCC. Notably, the proportion of SRCC was significantly higher among GC patients with bone metastasis compared to those without bone involvement (27.75% vs. 22.44%, *p* < 0.0001) (Figure S2a). Additionally, SRCC was more prevalent in patients with bone metastasis than in those with metastases to other sites (27.75% vs. 9.70%, *p* < 0.0001) (Figure S2b). Among stage IV GC patients, those with SRCC were significantly more likely to develop bone metastasis compared to their non‐SRCC counterparts (49.63% vs. 21.60%, *p* < 0.0001) (Figure S2c). These results highlight the distinct metastasis pattern of SRCC, particularly its higher propensity for bone metastasis, which is less commonly observed in other histological subtypes of gastric cancer.

### 3.3 Clinicopathological Features of Bone‐Metastatic PCC Compared With Non‐PCC Patients

Given the higher incidence of bone metastasis in PCC patients, we further explored the clinicopathological differences between bone‐metastatic PCC and non‐PCC patients (Table 2). PCC patients were significantly younger, with 58.18% under the age of 60, compared to 36.92% in the non‐PCC group (*p* = 0.020). Histologically, PCC was more frequently associated with diffuse‐type tumors (74.07% vs. 30.43%, *p* = 0.002). Although there was no significant difference in the bone metastatic phase between the two groups, metachronous metastasis was slightly more common in PCC patients (76.36% vs. 64.62%, *p* = 0.162). Biomarker analysis revealed significant molecular distinctions: PCC patients had lower Her‐2 positivity (2.08% vs. 25.00%, *p* = 0.001) and a higher rate of E‐cadherin negativity (23.08% vs. 0.00%, *p* = 0.049), indicating unique molecular characteristics compared to non‐PCC patients. Additionally, PD‐L1 expression was higher in non‐PCC patients, with 35.00% showing a CPS ≥ 10 compared to 11.43% in PCC patients (*p* = 0.042). At the time of bone metastasis diagnosis, PCC patients had lower neutrophil counts (*p* = 0.049) and neutrophil‐to‐lymphocyte ratio (NLR) (*p* = 0.002) but higher lymphocyte counts (*p* = 0.059) and lymphocyte‐to‐monocyte ratio (LMR) (*p* = 0.024) compared to non‐PCC patients (Table S2). These results suggest that bone‐metastatic PCC is associated with distinct clinicopathological features and immune profiles, which may closely relate to treatment responses and patient prognosis.

**TABLE 2:** Clinicopathological features of bone‐metastatic PCC compared with non‐PCC patients.

Characteristics	Total (*n* = 120)	non‐PCC (*n* = 65)	PCC (*n* = 55)	Statistic	*p*
Age (years), *n* (%)				χ^2^ = 5.41	**0.020**
< 60	56 (46.67)	24 (36.92)	32 (58.18)		
≥ 60	64 (53.33)	41 (63.08)	23 (41.82)		
Gender, *n* (%)				χ^2^ = 0.90	0.343
Male	71 (59.17)	41 (63.08)	30 (54.55)		
Female	49 (40.83)	24 (36.92)	25 (45.45)		
Lauren classification^a^, *n* (%)				χ^2^ = 12.50	**0.002**
Intestinal type	10 (20.00)	9 (39.13)	1 (3.70)		
Diffuse type	27 (54.00)	7 (30.43)	20 (74.07)		
Mixture	13 (26.00)	7 (30.43)	6 (22.22)		
Metastatic site, *n* (%)				χ^2^ = 0.13	0.719
Bone‐only metastasis	16 (13.33)	8 (12.31)	8 (14.55)		
Multiple metastases	104 (86.67)	57 (87.69)	47 (85.45)		
Bone metastatic phase, *n* (%)				χ^2^ = 1.96	0.162
Synchronous	36 (30.00)	23 (35.38)	13 (23.64)		
Metachronous	84 (70.00)	42 (64.62)	42 (76.36)		
Her‐2^a^, *n* (%)				χ^2^ = 10.77	**0.001**
+++, ++ with FISH+	13 (13.54)	12 (25.00)	1 (2.08)		
‐, +, ++	83 (86.46)	36 (75.00)	47 (97.92)		
VEGFR2^a^, *n* (%)				χ^2^ = 0.12	0.732
Positive	31 (64.58)	13 (61.90)	18 (66.67)		
Negative	17 (35.42)	8 (38.10)	9 (33.33)		
E‐cadherin^a^, *n* (%)				χ^2^ = 3.88	**0.049**
Positive	42 (87.50)	22 (100.00)	20 (76.92)		
Negative	6 (12.50)	0 (0.00)	6 (23.08)		
MMR status^a^, *n* (%)				χ^2^ = 0.00	1.000
dMMR	4 (5.56)	2 (5.41)	2 (5.71)		
pMMR	68 (94.44)	35 (94.59)	33 (94.29)		
PD‐L1^a^, *n* (%)				χ^2^ = 6.35	**0.042**
CPS < 1	34 (45.33)	14 (35.00)	20 (57.14)		
1 ≤ CPS < 10	23 (30.67)	12 (30.00)	11 (31.43)		
CPS ≥ 10	18 (24.00)	14 (35.00)	4 (11.43)		

Abbreviation: CPS: combined positive score. Bold values are statistically significant.

^a^ Indicates missing data for certain patients in the respective categories. These missing entries are due to incomplete records.

### 3.4 Survival Analysis of GC Patients With Bone Metastasis

**FIGURE 2:**
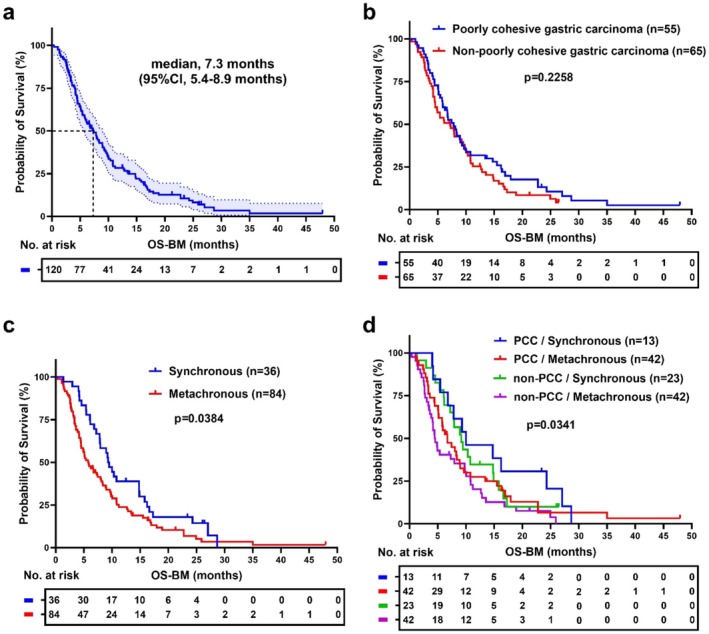
Survival analysis of GC patients from Nanjing Drum Tower Hospital. (a) Overall survival after bone metastasis (OS‐BM) in all GC patients with bone metastasis (*n* = 120). (b) Comparison of OS‐BM between poorly cohesive gastric carcinoma (PCC, *n* = 55) and non‐poorly cohesive gastric carcinoma (non‐PCC, *n* = 65) patients, *p* = 0.2258. (c) Comparison of OS‐BM between synchronous (*n* = 36) and metachronous (*n* = 84) bone metastasis in GC patients, *p* = 0.0384. (d) Subgroup analysis of OS‐BM by pathological type and bone metastatic phase, *p* = 0.0341. Kaplan–Meier survival analysis was performed using the log‐rank test with *p*‐values labeled.

After analyzing the clinicopathological characteristics of GC patients with bone metastasis, we focused on their survival outcomes. The median overall survival after bone metastasis (OS‐BM) was 7.3 months (95% CI: 5.4–8.9 months) (Figure 2a). A survival analysis comparing OS‐BM in PCC and non‐PCC patients showed no significant difference between the two groups (*p* = 0.2258) (Figure 2b). Both groups displayed similar survival patterns, with a median survival of 7.8 months (95% CI: 5.8–9.6 months) for PCC patients and 7.2 months (95% CI: 4.5–9.5 months) for non‐PCC patients. However, a significant difference in OS‐BM was observed when comparing synchronous and metachronous bone metastasis (*p* = 0.0384) (Figure 2c). This prompted a detailed subgroup analysis that revealed the bone metastatic phase significantly impacts survival outcomes (*p* = 0.0341). Specifically, patients with synchronous bone metastasis had longer OS‐BM compared to those with metachronous metastasis, a pattern consistent across both PCC and non‐PCC groups (Figure 2d).

### 3.5 Prognostic Factors Identified in GC Patients With Bone Metastasis

To further investigate factors influencing survival in GC patients with bone metastasis, we performed univariate and multivariate Cox regression analyses to identify both protective and adverse prognostic factors. As detailed in Table 3, the univariate analysis revealed several factors associated with poorer survival outcomes, including metachronous bone metastasis (*p* = 0.040), elevated CEA (≥ 10 ng/mL, *p* = 0.014), CA125 (≥ 30.2 U/mL, *p* = 0.002), WBC (≥  9.5 × 10^9^/L, *p* = 0.046), and LDH levels (≥ 245 U/L, *p* = 0.005). On the other hand, immunotherapy demonstrated a significant protective effect (*p* < 0.001). In the multivariate analysis, metachronous bone metastasis (HR = 2.35, 95% CI: 1.47–3.74, *p* < 0.001), elevated CA125 (HR = 1.60, 95% CI: 1.03–2.48, *p* = 0.036), and elevated WBC levels (HR = 2.15, 95% CI: 1.17–3.92, *p* = 0.013) were identified as independent predictors of poorer survival. Conversely, immunotherapy was independently associated with significantly improved survival outcomes (HR = 0.44, 95% CI: 0.29–0.66, *p* < 0.001).

**TABLE 3:** Univariate and multivariate Cox regression analysis of prognostic factors for OS‐BM of GC patients with bone metastases.

Variables	Univariate analysis					Multivariate analysis				
	*β*	S.E	*z*	*p*	HR (95% CI)	*β*	S.E	*z*	*p*	HR (95% CI)
Age (years)										
< 60					1.00 (Reference)					
≥ 60	0.32	0.20	1.60	0.110	1.37 (0.93 ~ 2.02)					
Gender										
Male					1.00 (Reference)					
Female	0.02	0.20	0.12	0.906	1.02 (0.70 ~ 1.50)					
Histological type										
non‐PCC					1.00 (Reference)					
PCC	−0.24	0.20	−1.21	0.228	0.79 (0.54 ~ 1.16)					
Metastatic site										
Bone‐only metastasis					1.00 (Reference)					
Multiple metastases	−0.12	0.28	−0.43	0.668	0.89 (0.51 ~ 1.53)					
Bone metastatic phase										
Synchronous					1.00 (Reference)					1.00 (Reference)
Metachronous	0.43	0.21	2.05	**0.040**	1.54 (1.02 ~ 2.33)	0.85	0.24	3.59	**< 0.001**	2.35 (1.47 ~ 3.74)
Her‐2										
‐, +, ++					1.00 (Reference)					
+++, ++ with FISH+	−0.32	0.31	−1.02	0.309	0.73 (0.39 ~ 1.34)					
VEGFR2										
Negative					1.00 (Reference)					
Positive	−0.30	0.32	−0.92	0.358	0.74 (0.40 ~ 1.40)					
E‐cadherin										
Negative					1.00 (Reference)					
Positive	−0.34	0.45	−0.75	0.451	0.71 (0.30 ~ 1.72)					
MMR status										
pMMR					1.00 (Reference)					
dMMR	0.70	0.53	1.32	0.186	2.01 (0.71 ~ 5.67)					
PDL1										
CPS < 1					1.00 (Reference)					
1 ≤ CPS < 10	−0.24	0.28	−0.84	0.403	0.79 (0.45 ~ 1.37)					
CPS ≥ 10	0.08	0.31	0.26	0.794	1.08 (0.59 ~ 2.00)					
CEA (ng/mL)										
< 10					1.00 (Reference)					1.00 (Reference)
≥ 10	0.48	0.20	2.45	**0.014**	1.61 (1.10 ~ 2.37)	0.38	0.24	1.60	0.110	1.46 (0.92 ~ 2.32)
CA19‐9 (U/L)										
< 27					1.00 (Reference)					
≥ 27	0.18	0.20	0.93	0.354	1.20 (0.82 ~ 1.76)					
CA125 (U/mL)										
< 30.2					1.00 (Reference)					1.00 (Reference)
≥ 30.2	0.61	0.19	3.13	**0.002**	1.84 (1.26 ~ 2.68)	0.47	0.22	2.09	**0.036**	1.60 (1.03 ~ 2.48)
CA242 (IU/mL)										
< 10					1.00 (Reference)					
≥ 10	0.21	0.20	1.07	0.284	1.23 (0.84 ~ 1.81)					
CA724 (Ug/L)										
< 6.9					1.00 (Reference)					
≥ 6.9	0.10	0.20	0.47	0.642	1.10 (0.74 ~ 1.64)					
AFP (Ug/L)										
< 10					1.00 (Reference)					
≥ 10	0.31	0.33	0.93	0.351	1.36 (0.71 ~ 2.62)					
WBC (×10^9^/L)										
< 9.5					1.00 (Reference)					1.00 (Reference)
≥ 9.5	0.54	0.27	1.99	**0.046**	1.72 (1.01 ~ 2.94)	0.76	0.31	2.48	**0.013**	2.15 (1.17 ~ 3.92)
Neutrophil (×10^9^/L)										
< 6.3					1.00 (Reference)					
≥ 6.3	0.40	0.24	1.67	0.095	1.49 (0.93 ~ 2.39)					
PLT (×10^9^/L)										
< 125					1.00 (Reference)					
≥ 125	−0.23	0.25	−0.92	0.358	0.79 (0.48 ~ 1.30)					
HB (g/L)										
< 120					1.00 (Reference)					
≥ 120	−0.00	0.20	−0.01	0.993	1.00 (0.68 ~ 1.46)					
LDH (U/L)										
< 245					1.00 (Reference)					1.00 (Reference)
≥ 245	0.54	0.19	2.80	**0.005**	1.72 (1.18 ~ 2.52)	0.38	0.22	1.75	0.080	1.46 (0.96 ~ 2.24)
ALP (U/L)										
< 185					1.00 (Reference)					
≥ 185	0.31	0.19	1.57	0.116	1.36 (0.93 ~ 1.99)					
Albumin (g/L)										
< 40					1.00 (Reference)					
≥ 40	−0.16	0.19	−0.80	0.423	0.86 (0.59 ~ 1.25)					
CRP (mg/L)										
< 8					1.00 (Reference)					
≥ 8	0.17	0.19	0.90	0.366	1.19 (0.82 ~ 1.73)					
Ca^2+^ (mmol/L)										
< 2.25					1.00 (Reference)					
≥ 2.25	0.14	0.20	0.70	0.483	1.15 (0.78 ~ 1.70)					
With immunotherapy										
No					1.00 (Reference)					1.00 (Reference)
Yes	−0.73	0.20	−3.69	**< 0.001**	0.48 (0.33 ~ 0.71)	−0.81	0.21	−3.93	**< 0.001**	0.44 (0.29 ~ 0.66)
With anti‐angiogenic therapy										
No					1.00 (Reference)					
Yes	0.06	0.19	0.29	0.769	1.06 (0.72 ~ 1.55)					
With local radiotherapy for BM										
No					1.00 (Reference)					
Yes	−0.28	0.23	−1.20	0.231	0.76 (0.48 ~ 1.20)					

Abbreviations: CI: confidence interval; HR: hazard ratio; S.E: standard error. Bold values are statistically significant.

**FIGURE 3:**
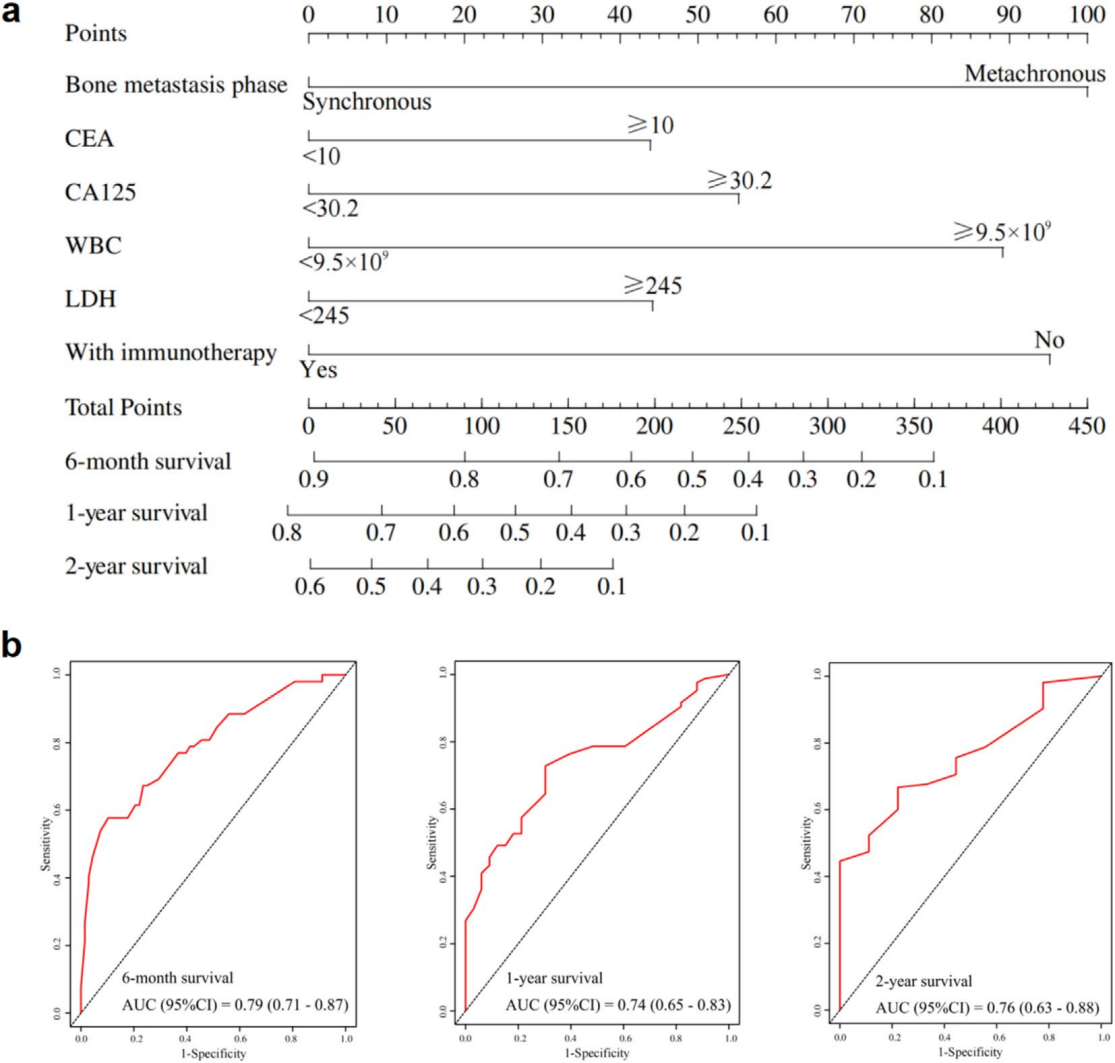
Prognostic nomogram and validation for GC patients with bone metastasis. (a) Nomogram constructed using significant prognostic factors identified from Cox regression analysis to predict 6‐month, 1‐year, and 2‐year survival probabilities. (b) ROC curves assessing the nomogram's predictive accuracy for 6‐month, 1‐year, and 2‐year survival.

To further evaluate whether these prognostic factors could effectively predict survival outcomes in GC patients with bone metastasis, we incorporated variables with *p* < 0.05 from the Cox regression analysis into a prognostic nomogram. Each variable was assigned a score by projecting onto an upward scale, and the total scores were used to estimate survival probabilities at 6 months, 1 year, and 2 years (Figure 3a). The nomogram demonstrated strong discriminatory power, with AUC values of 0.79 (95% CI: 0.71–0.87) for 6‐month survival, 0.74 (95% CI: 0.65–0.83) for 1‐year survival, and 0.76 (95% CI: 0.63–0.88) for 2‐year survival (Figure 3b). Therefore, this model could serve as a valuable tool for predicting survival outcomes in GC patients with bone metastasis, helping clinicians identify high‐risk patients who may benefit from more intensive monitoring or personalized treatment strategies.

### 3.6 Survival Benefit of Immunotherapy in Various Sub‐Groups of Bone‐Metastatic GC Patients

**FIGURE 4:**
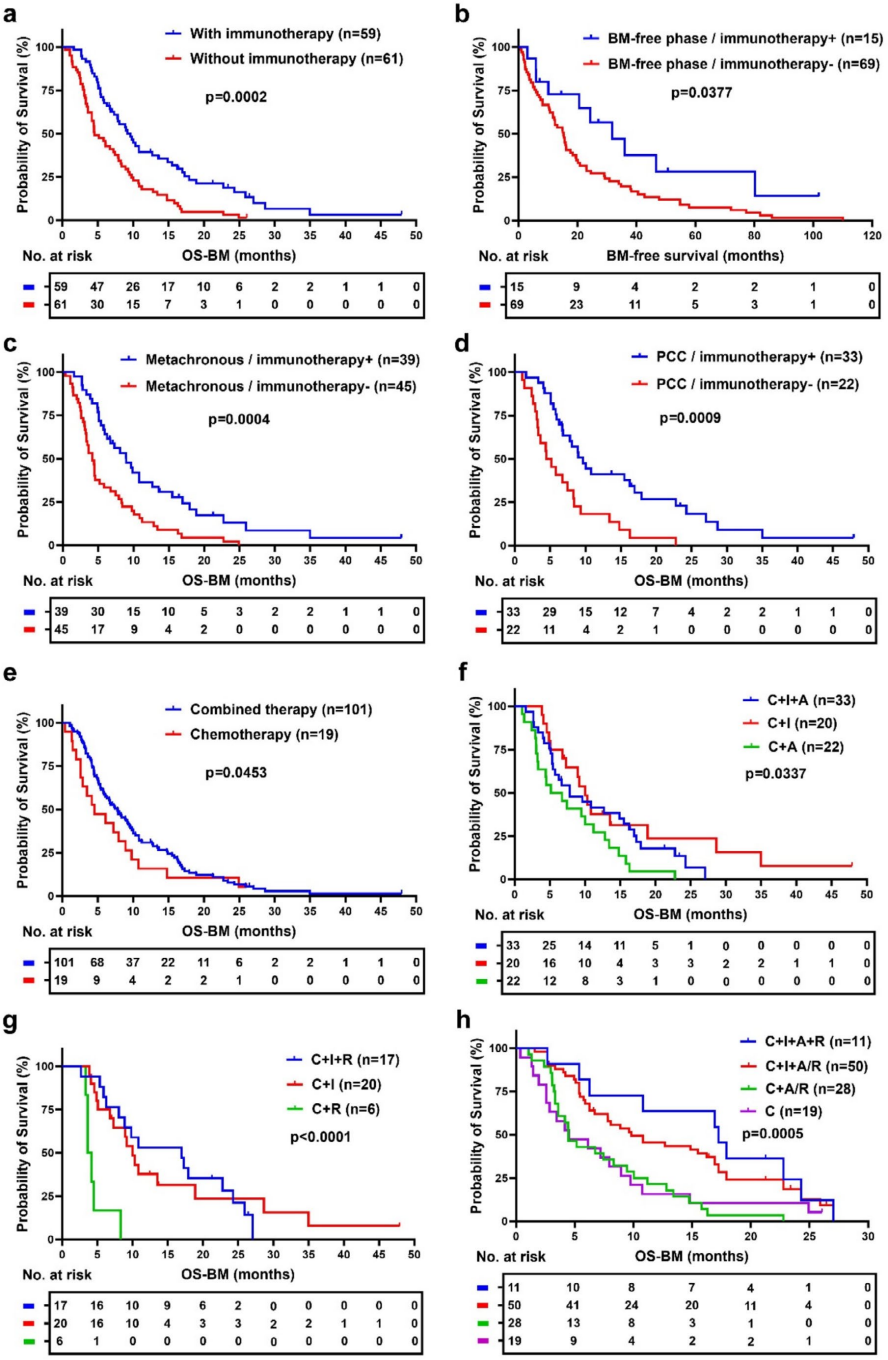
Survival analysis of different treatment strategies in GC patients with bone metastasis. (a–d) Survival benefit of immunotherapy in various sub‐groups of bone‐metastatic gastric cancer (GC) patients, all of whom received chemotherapy. (a) Kaplan–Meier survival curve comparing overall survival after bone metastasis (OS‐BM) between patients who received immunotherapy and those who did not, *p* = 0.0002. (b) Bone metastasis‐free survival in patients who received immunotherapy before the onset of bone metastasis compared to those who did not, *p* = 0.0377. (c) OS‐BM for patients with metachronous bone metastasis receiving immunotherapy versus those who did not, *p* = 0.0004. (d) OS‐BM for patients with poorly cohesive gastric carcinoma (PCC) receiving immunotherapy compared to those who did not, *p* = 0.0009. (e–h) Survival benefit of combinational therapies in bone‐metastatic GC patients, all of whom received chemotherapy. (e) OS‐BM between patients receiving combined therapy and those receiving chemotherapy alone, *p* = 0.0453. (f) OS‐BM for patients receiving immunotherapy combined with anti‐angiogenic therapy versus those receiving either therapy alone, *p* = 0.0070. C + I + A: Chemotherapy combined with immunotherapy and anti‐angiogenic therapy; C + I: Chemotherapy combined with immunotherapy; C + A: Chemotherapy combined with anti‐angiogenic therapy. (g) OS‐BM for patients receiving immunotherapy with local radiotherapy compared to those receiving either treatment alone, *p* < 0.0001. C + I + R: Chemotherapy combined with immunotherapy and local radiotherapy; C + I: Chemotherapy combined with immunotherapy; C + R: Chemotherapy combined with local radiotherapy. (h) OS‐BM among patients receiving immunotherapy combined with anti‐angiogenic therapy and/or local radiotherapy, those receiving anti‐angiogenic therapy or local radiotherapy (without immunotherapy), and those receiving chemotherapy alone, *p* = 0.0005. C + I + A + R: Chemotherapy combined with immunotherapy, anti‐angiogenic therapy, and local radiotherapy; C + I + A/R: Chemotherapy combined with immunotherapy and either anti‐angiogenic therapy or local radiotherapy; C + A/R: Chemotherapy combined with anti‐angiogenic therapy or local radiotherapy; C: Chemotherapy alone. Statistical analyses were performed using log‐rank test with *p*‐values labeled.

After identifying immunotherapy as an independent favorable prognostic factor, we conducted a detailed survival analysis to assess its influence on patient outcomes. As shown in Figure 4a, patients who received immunotherapy had significantly longer survival compared to those who did not (*p* = 0.0002), with a median overall survival after bone metastasis (OS‐BM) of 9.63 months versus 4.53 months, respectively. Additionally, we explored whether preemptive immunotherapy administered before the onset of bone metastasis could prolong bone metastasis‐free survival (BMFS) in advanced gastric cancer patients. Our analysis showed that patients receiving immunotherapy before bone metastasis had a significantly extended BMFS compared to those who did not (31.73 months vs. 15.27 months, *p* = 0.0377) (Figure 4b).

To further determine which subgroups of GC patients with bone metastasis benefit most from immunotherapy, we applied a Cox regression model to estimate hazard ratios (HRs) and 95% confidence intervals (CIs) across various patient subgroups, as illustrated in the forest plot (Figure S3). Subgroup analysis demonstrated that the survival advantage provided by immunotherapy was consistent across multiple clinical and pathological factors. For instance, patients with elevated tumor markers such as CA125 (≥ 30.2 U/mL) and CEA (≥ 10 ng/mL) experienced notable survival improvements when immunotherapy was included in their treatment regimen (HR = 1.97, 95% CI: 1.10–3.29, and HR = 2.50, 95% CI: 1.45–4.32, respectively). Furthermore, patients with PCC and those with metachronous bone metastasis showed significant reductions in mortality risk when treated with immunotherapy (HR = 2.64, 95% CI: 1.35–5.16, *p* = 0.013, and HR = 2.24, 95% CI: 1.34–3.56, *p* < 0.001, respectively). Kaplan–Meier survival analysis further confirmed these findings. Patients with metachronous bone metastasis who received immunotherapy had significantly longer OS‐BM compared to those who did not (8.97 months vs. 4.17 months, *p* = 0.0004) (Figure 4c). Similarly, among PCC patients, those treated with immunotherapy exhibited a marked extension in OS‐BM (9.63 months vs. 4.82 months, *p* = 0.0009) (Figure 4d). Additionally, patients with specific tumor molecular features, including Her‐2 negativity, VEGFR2 positivity, E‐cadherin positivity, and pMMR, showed a more pronounced survival benefit from immunotherapy (Figure S3). These results suggest that tumor molecular profiling could serve as a valuable tool for identifying patients most likely to benefit from immunotherapy.

Moreover, further analysis revealed that other treatment strategies, such as anti‐angiogenic therapy and local radiotherapy for BM, did not significantly extend survival time (Figure S4). Beyond the overall analysis, we examined different subgroups, such as patients with PCC and those with metachronous bone metastasis, and found that they did not derive significant survival benefits from these treatments (Figures S5 and S6). Taken together, these results highlight the potential of immunotherapy to improve survival outcomes in high‐risk subgroups of GC patients with bone metastasis, especially those with PCC or metachronous metastasis.

### 3.7 Survival Benefit of Combinational Therapies in GC Patients With Bone Metastasis

Given the observed survival benefits of immunotherapy in multiple subgroups of GC patients with bone metastasis, we next explored whether combining different treatment strategies could further enhance survival outcomes. Our results showed that patients receiving combined therapies had significantly longer overall survival after bone metastasis (OS‐BM) compared to those receiving chemotherapy alone (7.80 months vs. 4.53 months, *p* = 0.0453) (Figure 4e). To investigate the specific impact of different combinations, we stratified patients based on their treatment regimens, which included immunotherapy, anti‐angiogenic therapy, and local radiotherapy for bone metastasis. Survival analysis revealed that combining immunotherapy with either anti‐angiogenic therapy or local radiotherapy provided the greatest survival benefits. Specifically, patients treated with both immunotherapy and anti‐angiogenic therapy had significantly longer OS‐BM compared to those who received anti‐angiogenic therapy alone (7.83 months vs. 5.95 months, *p* = 0.0337) (Figure 4f). Similarly, combining immunotherapy with local radiotherapy significantly extended survival compared to either treatment alone (16.93 months vs. 10.00 months vs. 3.88 months, *p* < 0.0001) (Figure 4g). In a similar vein, patients treated with both immunotherapy, anti‐angiogenic therapy, and local radiotherapy showed a notable enhancement in OS‐BM compared to those receiving either anti‐angiogenic therapy or local radiotherapy (without immunotherapy), or chemotherapy alone, with survival times significantly extended (17.27 months vs. 4.47 months vs. 4.53 months, *p* = 0.0005) (Figure 4h). Collectively, these findings support the use of combinational therapies, particularly those incorporating immunotherapy, as a standard strategy for managing GC patients with bone metastasis.

## 4 Discussion

Bone metastasis remains an incurable form of cancer with extremely poor prognosis and increasing incidence in GC [2, 7, 8]. However, the comprehensive analysis of clinicopathological characteristics, prognostic factors, and treatment strategies for bone‐metastatic GC patients is still inadequate. Our results revealed that PCC patients have a higher incidence of bone metastasis compared to non‐PCC patients. Additionally, bone‐metastatic PCC patients exhibited distinct clinicopathological characteristics, including lower expression of E‐cadherin, Her‐2, and PD‐L1. We also identified independent prognostic factors influencing OS‐BM, including immunotherapy, metachronous bone metastasis, elevated tumor markers (such as CEA and CA125), and increased WBC and LDH levels. As for treatment, although clinical and preclinical studies have shown that patients with bone metastases from cancers such as lung and breast cancer fail to benefit from immunotherapy, we found that immunotherapy brought survival benefits in bone‐metastatic GC patients, with further improved patient survival observed when combined with anti‐angiogenic therapy or local radiotherapy.

Consistent with previous studies [11, 32, 33, 34], our analysis of the SEER database confirmed that SRCC patients are significantly more prone to developing bone metastasis than non‐SRCC patients, with a higher incidence of bone metastasis than visceral metastasis. However, the absence of peritoneal metastasis data within the SEER database prevents direct comparisons, indicating the need for future studies to fully elucidate the metastatic patterns of SRCC. The high incidence of bone metastasis in PCC, particularly SRCC, is likely driven by its distinct clinicopathological features, including low E‐cadherin expression, which may partly explain the increased metastatic potential. The Wnt/β‐catenin signaling pathway, which plays a critical role in regulating the bone microenvironment, has been shown to promote bone metastasis [35, 36]. Reduced E‐cadherin expression, commonly seen in PCC, leads to elevated levels of cytoplasmic β‐catenin, which subsequently activates downstream signaling pathways that drive bone metastasis [37, 38, 39]. Additionally, mutations in the CDH1 gene, which encodes E‐cadherin, are frequently observed in PCC and have been linked to the aggressive nature of this subtype [40, 41]. The unique molecular characteristics of PCC, including high expression of mesenchymal markers such as USP51, ZEB1, and ACTA2, further induce E‐cadherin deficiency and enhance its metastatic potential [42, 43]. Additionally, our comparative analysis showed that PCC patients with bone metastasis are generally younger and have a higher proportion of poorly differentiated, diffuse‐type tumors. These patients also tend to develop metachronous bone metastasis, which occurs after the initial diagnosis of gastric cancer. These findings provide valuable insights into the unique clinicopathological characteristics of PCC, shedding light on the mechanisms behind its aggressive behavior and propensity for bone metastasis.

In this study, we identified elevated CEA, CA125, and LDH levels as adverse prognostic factors of bone‐metastatic GC patients, which is consistent with findings from previous reports [5, 16, 32, 33, 34]. Additionally, a 2014 clinical study from Park et al. [16] indicated that elevated CA199 is also an adverse prognostic marker, while Kim et al. [5] found that thrombocytopenia, elevated ALP, and hypercalcemia are linked to poorer outcomes. These discrepancies likely reflect the heterogeneity of gastric cancer, emphasizing the need for further research to understand these associations. We also identified elevated WBC levels (≥ 9.5 × 10^9^/L) as an independent adverse prognostic factor. This could reflect local or systemic inflammatory responses triggered by bone metastasis, as both tumor and immune cells in the bone microenvironment release inflammatory mediators such as prostaglandins, interleukins, and tumor necrosis factors [44, 45, 46, 47]. These mediators promote granulocyte release from the bone marrow into the bloodstream, leading to elevated WBC counts and a heightened inflammatory state, which is often linked to worse prognoses. Additionally, metachronous bone metastasis emerged as an independent risk factor for poor survival outcomes. This may be attributed to the fact that patients with metachronous metastasis have typically undergone multiple lines of anti‐tumor treatment prior to the detection of bone metastasis. As the disease progresses through these treatments, the likelihood of developing resistance increases, making subsequent therapies less effective and complicating patient management. While several studies have suggested that factors such as tumor invasion depth and the extent of lymph node metastasis also play a role in prognosis [5, 16, 32], our study was limited by the lack of TNM staging data for many patients who did not undergo surgery, underscoring the need for further research with larger, more comprehensive datasets.

Several preclinical studies suggest that the bone microenvironment has immunosuppressive properties, which impede the efficacy of immunotherapies, such as immune checkpoint inhibitors (ICIs) [48, 49, 50]. Specifically, tumor cells in bone metastasis secrete cytokines that disrupt bone remodeling by stimulating osteoblast and osteoclast activity, releasing pro‐tumor factors from the bone matrix, and impairing immune function [51, 52]. For example, TGF‐β released by osteoclasts promotes suppressive Th17 polarization and inhibits Th1 effector T cell development, thereby weakening T cell‐mediated antitumor responses [53]. In addition, Tregs further exacerbate this immunosuppressive environment by facilitating the recruitment of disseminated tumor cells to the bone and promoting immune evasion [54]. Furthermore, tumor cells within the bone microenvironment interfere with key ligand‐receptor interactions (NKR/NKR‐L and TRAIL/DR4) and activate TAM tyrosine kinase receptors, which collectively impair NK cell antitumor activity [55, 56]. Meanwhile, MDSCs play a crucial role by suppressing T cell function and promoting Treg expansion. As tumor progression continues, MDSCs accumulate in the bone, where they differentiate into osteoclasts, contributing to bone destruction [57]. Taken together, these factors create a highly immunosuppressive microenvironment, significantly diminishing the response of solid tumors, such as breast and lung cancer, to immunotherapy. Nevertheless, our study found that immunotherapy significantly extended the survival of GC patients with bone metastasis, suggesting that ICIs may have a therapeutic effect on bone lesions, at least in the context of GC. More research efforts are needed to further explore the potential mechanisms through which immunotherapy benefits bone‐metastatic GC patients.

Our study suggests that the PCC subtype is particularly prone to bone metastasis due to its distinct pathological characteristics, emphasizing the need to delay metastasis and prolong survival in advanced PCC patients. Single‐cell analysis has shown that PCC harbors a unique immunosuppressive microenvironment, characterized by increased infiltration of follicular B cells and Tregs, alongside reduced levels of CD8^+^ effector T cells, which diminishes the response to immunotherapy [12, 13]. However, clinical studies have demonstrated that immunotherapy can significantly improve survival in advanced PCC patients, especially in those with an Eastern Cooperative Oncology Group (ECOG) performance status of 0 and high PD‐L1 expression [58]. Our findings align with these studies, showing that PCC patients with bone metastasis derive substantial benefits from immunotherapy, with the median OS‐BM extending from 4.82 months to 9.63 months in patients receiving immunotherapy. Furthermore, PCC patients exhibited a lower NLR and a higher LMR, both of which are key prognostic indicators that may correlate with the observed improvements in survival. These immunological markers may serve as potential predictors of immunotherapy efficacy in PCC, though further large‐scale studies are required to validate these findings. Given PCC's tendency to develop metachronous bone metastasis (a known indicator of poor prognosis) and the potential of immunotherapy to extend the bone metastasis‐free survival period, our study underscores the importance of early application of immunotherapy in advanced PCC patients. Early intervention with immunotherapy could delay the onset of bone metastasis and improve overall survival, offering a more favorable prognosis for this high‐risk group.

Several studies have shown that conventional treatments for bone metastasis, including chemotherapy, bone‐targeting agents, anti‐angiogenic therapies, and local radiotherapy, can improve the immune microenvironment [59, 60, 61, 62]. Building on this, combining treatment strategies including immunotherapy and other interventions can leverage the improved immune microenvironment to further enhance therapeutic outcomes [63, 64, 65]. For example, bisphosphonates have been shown to inhibit osteoclast‐mediated bone resorption by suppressing the receptor activator of nuclear factor kappa‐B ligand (RANKL) pathway [61]. Certain chemotherapeutic agents can reduce Tregs and induce apoptosis in MDSCs [59, 60]. Additionally, anti‐angiogenic therapy has been found to normalize disorganized tumor blood vessels and reduce the recruitment of immune‐inhibitory factors such as IL‐6, IL‐10, and indoleamine 2,3‐dioxygenase (IDO), improving the efficacy of immunotherapy [63]. Radiotherapy can also enhance immunotherapy by releasing damage‐associated molecular patterns and cytokines from tumor cells, which stimulate antigen‐specific cytotoxic CD8^+^ T cells and normalize the tumor vascular system [64, 65]. Here, we found that patients who received both immunotherapy and anti‐angiogenic therapy or local radiotherapy had significantly longer survival than those who received these therapies alone. Therefore, by directly eliminating tumor cells and indirectly remodeling the immunosuppressive bone microenvironment, combinational strategies could serve as a better treatment option for GC patients with bone metastasis.

In summary, by conducting a comprehensive analysis, this study provides detailed information about the clinicopathological features and prognostic factors of bone‐metastatic GC patients, including a noticeable finding that PCC has a higher bone metastasis incidence. Moreover, our findings suggest that immunotherapy‐based combination therapies improve survival in bone‐metastatic GC patients, thus providing novel insights into the clinical management of GC populations with a high risk of bone metastasis.

## Author Contributions

**Shiji Ren:** data curation (lead), formal analysis (lead), investigation (lead), methodology (lead), visualization (lead), writing – original draft (lead). **Yutao Wei:** data curation (equal), investigation (equal). **Wenqi Liu:** data curation (equal), investigation (equal). **Yipeng Zhang:** data curation (supporting), formal analysis (supporting), methodology (supporting). **Yue Wang:** data curation (supporting), formal analysis (supporting). **Ju Yang:** data curation (supporting), project administration (supporting), resources (supporting). **Baorui Liu:** conceptualization (supporting), project administration (supporting), resources (supporting), supervision (supporting). **Tao Shi:** conceptualization (lead), funding acquisition (equal), methodology (equal), resources (equal), supervision (equal), writing – review and editing (equal). **Jia Wei:** conceptualization (lead), funding acquisition (lead), project administration (lead), resources (lead), supervision (lead), writing – review and editing (lead).

## Ethics Statement

All procedures were performed in accordance with the Helsinki Declaration of 1964 and later versions. This study was approved by the Ethics Committee of Nanjing University Medical School Affiliated Drum Tower Hospital (No. 2021‐324‐01).

## Conflicts of Interest

The authors declare no conflicts of interest.

## Precis

Bone‐metastatic GC patients exhibit distinct clinicopathological features and prognostic factors. Immunotherapy‐based combinational treatments improve patient survival, indicating the benefits of immunotherapy in GC patients at high risk of bone metastasis.

## Supporting information


Data S1.

## Data Availability

The authors have nothing to report.
