# Supplementary material for: Clinical Characteristics, Prognostic Factors and Therapeutic Strategies in Gastric Cancer Patients With Bone Metastasis: A Retrospective Analysis
Source: Cancer Med. 2025 Mar 19;14(6):e70781. doi: 10.1002/cam4.70781 (PMC11921140; doi:10.1002/cam4.70781)
Supplement: Supplementary file 1 — Data S1. [file CAM4-14-e70781-s001.docx]

**Supplemental Materials for**

**Clinical characteristics, prognostic factors and therapeutic strategies in gastric cancer patients with bone metastasis: a retrospective analysis**

Shiji Ren^1^, Yutao Wei^2^, Wenqi Liu^3^, Yipeng Zhang^1^, Yue Wang^1^, Ju Yang^1^, Baorui Liu^1^, Tao Shi^1*^, Jia Wei^1,2,4*^


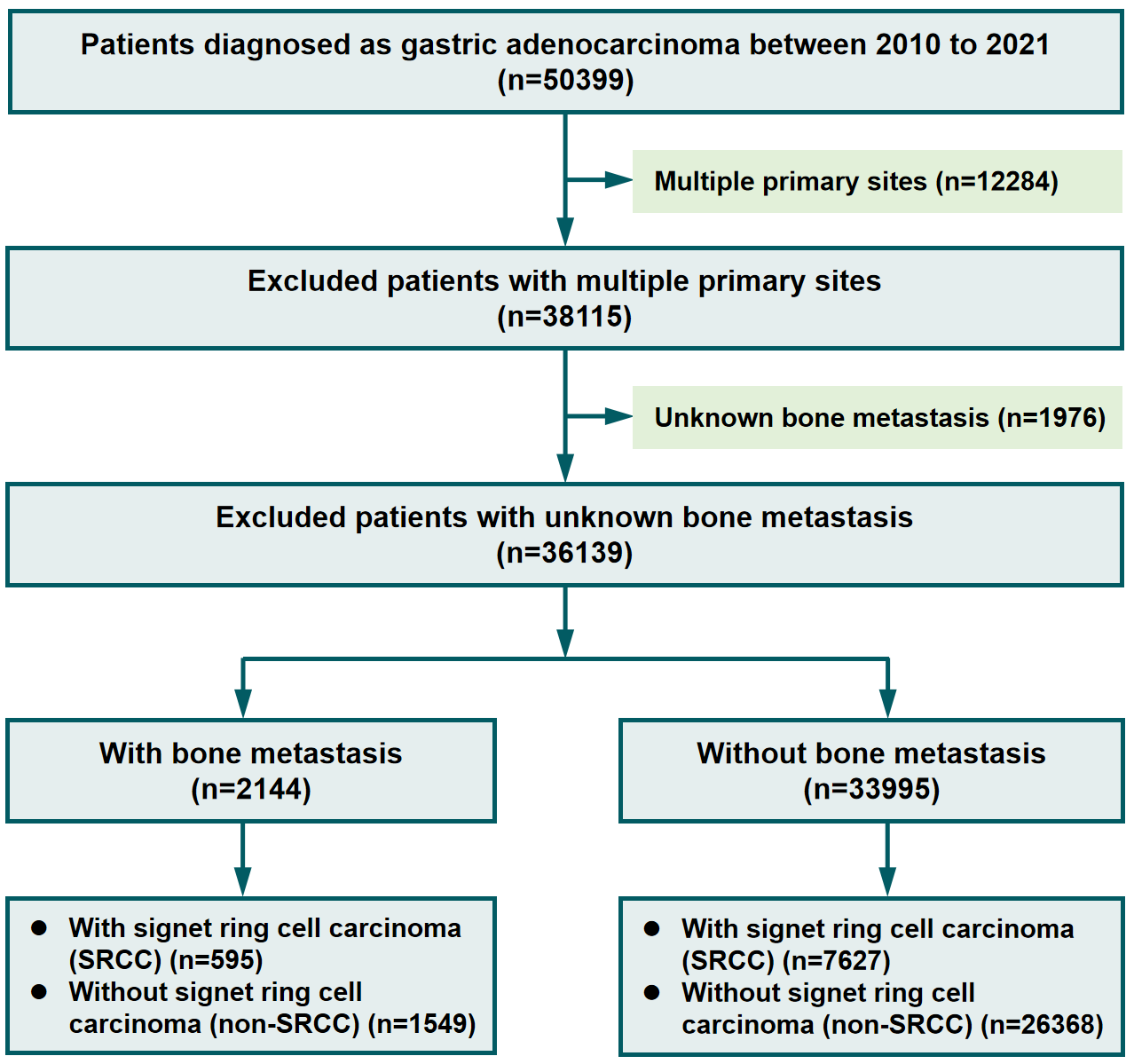


**Figure S1. The flowchart for data selection from the SEER database.** SRCC: signet ring cell carcinoma, a poorly cohesive gastric carcinoma (PCC) subtype defined by more than 90% poorly cohesive cells with signet ring cell morphology**.**


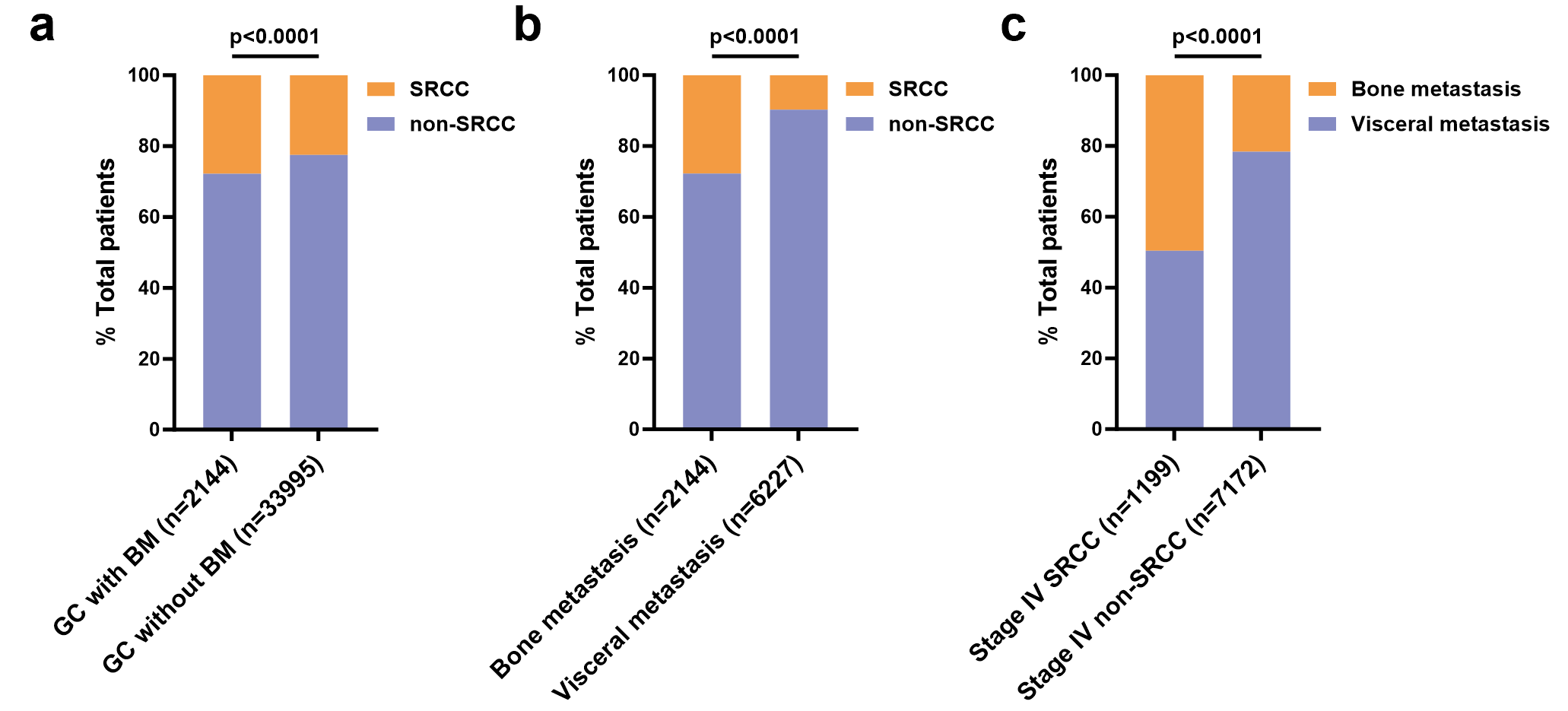


**Figure S2.** **Clinicopathological features of GC patients from the SEER database.** **(a)** Proportion of signet ring cell carcinoma (SRCC), a subtype of poorly cohesive gastric carcinoma (PCC) defined by more than 90% of the cells exhibiting signet ring cell morphology, in GC patients with and without bone metastasis. **(b)** Proportion of SRCC in GC patients with bone metastasis versus visceral metastasis (lung, liver, brain). **(c)** Incidence of bone metastasis in stage IV SRCC compared to non-SRCC patients. Statistical analysis was performed using Chi‐square tests (χ^2^ tests).

**
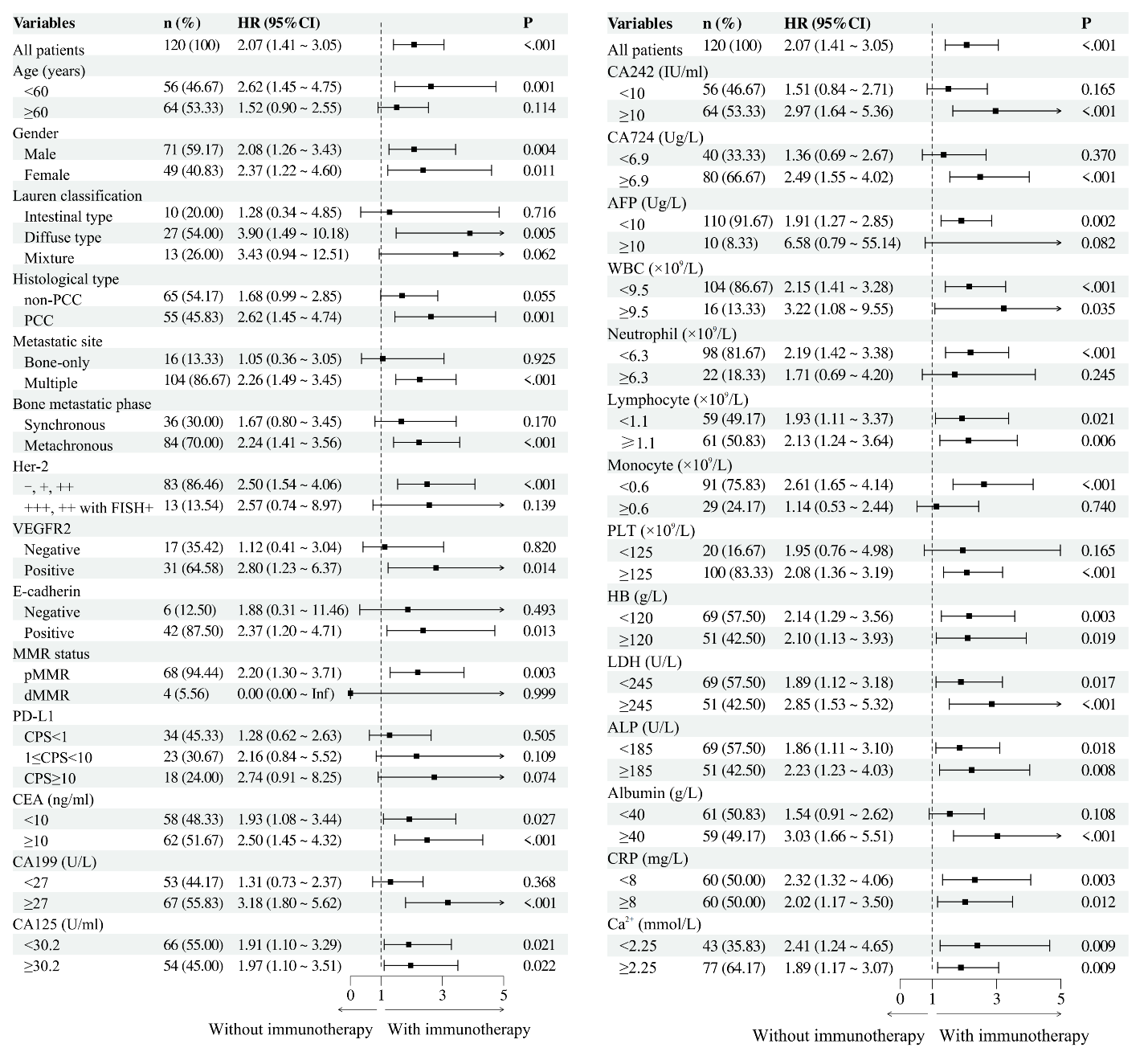
**

**Figure S3. Forest plot of hazard ratios (HRs) and 95% confidence intervals (CIs) from a Cox regression analysis evaluating the impact of immunotherapy on overall survival after bone metastasis (OS-BM) in GC patients with bone metastasis.**


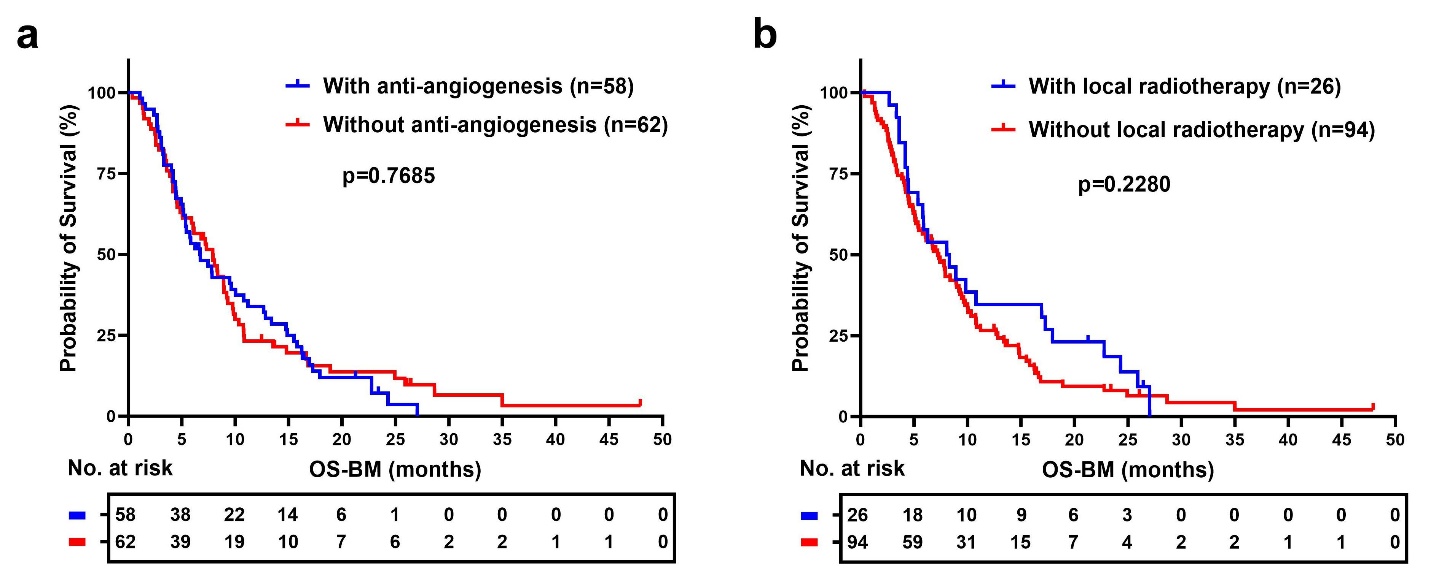


**Figure S4. Survival benefit of different treatment strategies in GC patients with bone metastasis.** **(a)** Kaplan-Meier survival curve comparing overall survival after bone metastasis (OS-BM) between patients who received anti-angiogenic therapy and those who did not, p=0.7685. **(b)** Kaplan-Meier survival curve comparing OS-BM between patients who received local radiotherapy and those who did not, p=0.2280. Kaplan-Meier survival analysis was performed using log-rank test with p values labeled.


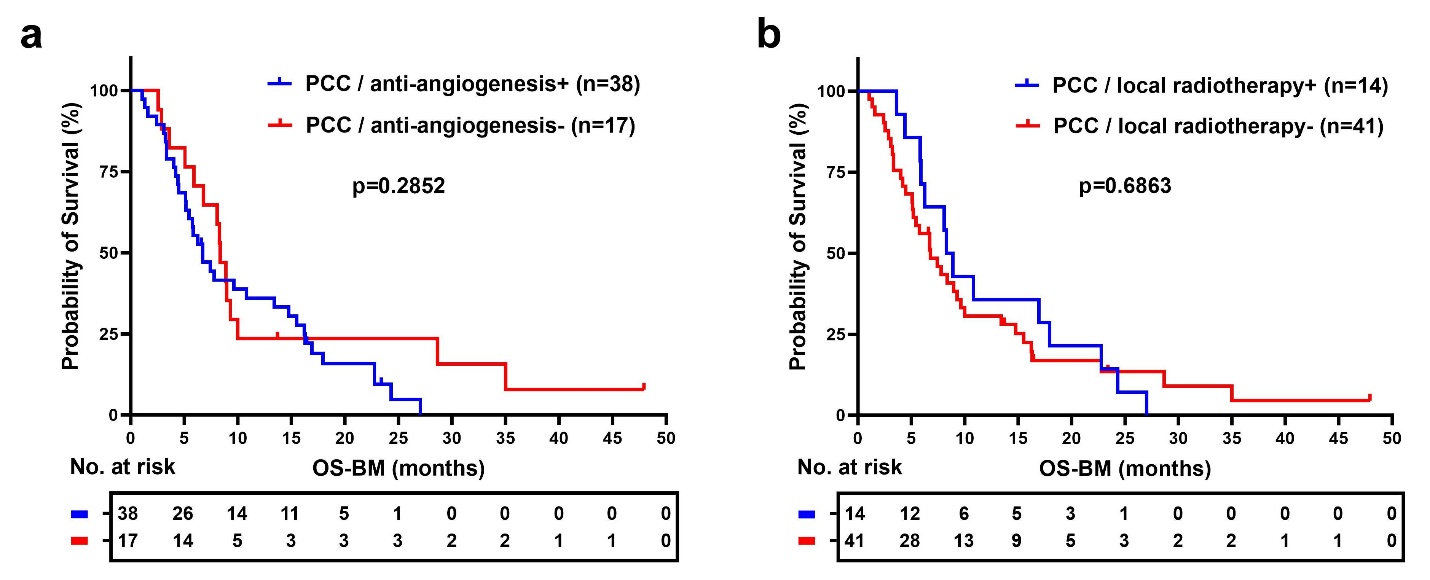


**Figure S5. Survival benefit of different treatment strategies in bone-metastatic poorly cohesive gastric carcinoma (PCC) patients.** **(a)** Kaplan-Meier survival curve comparing overall survival after bone metastasis (OS-BM) between bone-metastatic PCC patients who received anti-angiogenic therapy and those who did not, p=0.2852. **(b)** Kaplan-Meier survival curve comparing OS-BM between bone-metastatic PCC patients who received local radiotherapy and those who did not, p=0.6863. Kaplan-Meier survival analysis was performed using log-rank test with p values labeled.


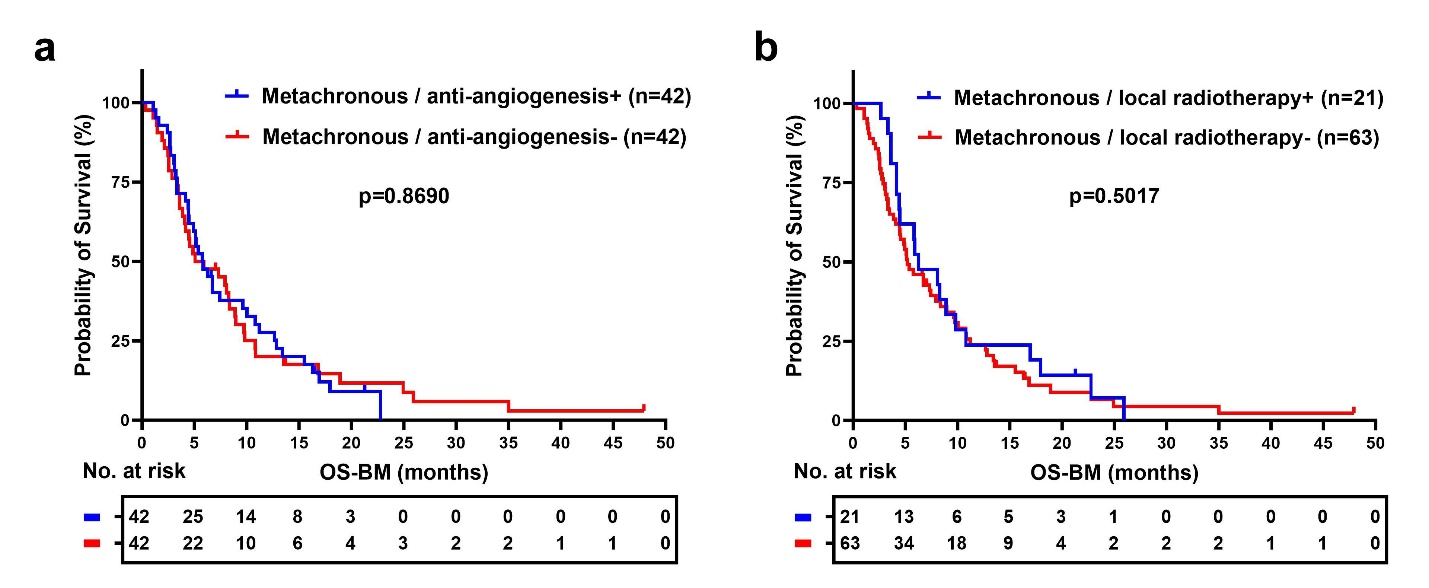


**Figure S6. Survival benefit of different treatment strategies in GC patients with metachronous bone metastasis.** **(a)** Kaplan-Meier survival curve comparing overall survival after bone metastasis (OS-BM) between metachronous bone-metastatic GC patients who received anti-angiogenic therapy and those who did not, p=0.8690. **(b)** Kaplan-Meier survival curve comparing OS-BM between metachronous bone-metastatic GC patients who received local radiotherapy and those who did not, p=0.5017. Kaplan-Meier survival analysis was performed using log-rank test with p values labeled.

**Table S1. Laboratory Data at the Time of Bone Metastasis Diagnosis in GC Patients.**

| **Laboratory data** | **Bone metastasis (n=120)** |
| --- | --- |
| CEA (ng/ml), M (Q₁, Q₃) | 12.36 (2.30, 78.17) |
| CA19-9 (U/L), M (Q₁, Q₃) | 37.78 (10.08, 514.75) |
| CA125 (U/ml), M (Q₁, Q₃) | 24.15 (11.43, 72.70) |
| CA242 (IU/ml), M (Q₁, Q₃) | 11.34 (3.91, 108.00) |
| CA724 (Ug/L), M (Q₁, Q₃) | 20.73 (4.13, 78.33) |
| AFP (Ug/L), M (Q₁, Q₃) | 2.70 (1.70, 4.43) |
| WBC (×10^9^/L), M (Q₁, Q₃) | 5.70 (4.30, 7.12) |
| Neutrophil (×10^9^/L), M (Q₁, Q₃) | 3.90 (2.98, 5.60) |
| Lymphocyte (×10^9^/L), M (Q₁, Q₃) | 1.10 (0.80, 1.50) |
| Monocyte (×10^9^/L), M (Q₁, Q₃) | 0.40 (0.30, 0.50) |
| PLT (×10^9^/L), M (Q₁, Q₃) | 184.00 (139.75, 229.00) |
| HB (g/L), M (Q₁, Q₃) | 116.50 (101.75, 127.25) |
| NLR, M (Q₁, Q₃) | 4.05 (2.69, 6.02) |
| LMR, M (Q₁, Q₃) | 2.67 (1.82, 4.00) |
| PLR, M (Q₁, Q₃) | 169.33 (117.04, 249.09) |
| LDH (U/L), M (Q₁, Q₃) | 231.00 (184.00, 322.25) |
| ALP (U/L), M (Q₁, Q₃) | 159.65 (92.00, 297.60) |
| Albumin (g/L), M (Q₁, Q₃) | 39.90 (35.98, 41.62) |
| CRP (mg/L), M (Q₁, Q₃) | 7.85 (3.40, 26.83) |
| Ca^2+^ (mmol/L), M (Q₁, Q₃) | 2.30 (2.19, 2.39) |

M: Median, Q₁: 1st Quartile, Q₃: 3st Quartile; NLR: neutrophil-to-lymphocyte ratio, PLR: platelet-to-lymphocyte ratio, LMR: lymphocyte-to-monocyte ratio

**Table S2. Laboratory Data of bone-metastatic PCC compared with non-PCC patients.**

| **Laboratory data, M (Q₁, Q₃)** | **Total**  **(n = 120)** | **non-PCC**  **(n = 65)** | **PCC**  **(n = 55)** | **Statistic** | ***P*** |
| --- | --- | --- | --- | --- | --- |
| CEA (ng/ml) | 12.36 (2.30, 78.17) | 19.07 (2.54, 100.00) | 5.75 (1.83, 47.81) | Z=-1.30 | 0.193 |
| CA19-9 (U/L) | 37.78 (10.08, 514.75) | 42.40 (7.63, 511.80) | 34.37 (13.05, 481.95) | Z=-0.45 | 0.656 |
| CA125 (U/ml) | 24.15 (11.43, 72.70) | 28.80 (12.90, 75.10) | 17.80 (11.10, 67.50) | Z=-1.10 | 0.273 |
| CA242 (IU/ml) | 11.34 (3.91, 108.00) | 15.71 (3.26, 139.20) | 9.47 (4.45, 101.77) | Z=-0.41 | 0.685 |
| CA724 (Ug/L) | 20.73 (4.13, 78.33) | 19.10 (3.13, 117.20) | 22.54 (6.46, 70.94) | Z=-0.67 | 0.505 |
| AFP (Ug/L) | 2.70 (1.70, 4.43) | 2.70 (1.50, 4.90) | 2.70 (1.90, 3.50) | Z=-0.10 | 0.918 |
| WBC (×10^9^/L) | 5.70 (4.30, 7.12) | 6.00 (4.40, 8.20) | 5.30 (4.30, 6.50) | Z=-1.63 | 0.104 |
| Neutrophil (×10^9^/L) | 3.90 (2.98, 5.60) | 4.40 (3.00, 5.80) | 3.50 (2.85, 4.55) | Z=-1.97 | **0.049** |
| Lymphocyte (×10^9^/L) | 1.10 (0.80, 1.50) | 1.00 (0.70, 1.20) | 1.10 (0.85, 1.50) | Z=-1.89 | 0.059 |
| Monocyte (×10^9^/L) | 0.40 (0.30, 0.50) | 0.40 (0.30, 0.60) | 0.40 (0.30, 0.50) | Z=-0.60 | 0.546 |
| PLT (×10^9^/L) | 184.00 (139.75, 229.00) | 169.00 (136.00, 229.00) | 194.00 (142.50, 224.50) | Z=-0.74 | 0.461 |
| HB (g/L) | 116.50 (101.75, 127.25) | 115.00 (102.00, 129.00) | 118.00 (101.50, 126.50) | Z=-0.47 | 0.637 |
| NLR | 4.05 (2.69, 6.02) | 4.67 (2.88, 7.00) | 3.55 (2.18, 4.91) | Z=-3.17 | **0.002** |
| LMR | 2.67 (1.82, 4.00) | 2.40 (1.71, 3.67) | 3.00 (2.00, 5.00) | Z=-2.26 | **0.024** |
| PLR | 169.33 (117.04, 249.09) | 176.67 (125.71, 250.00) | 154.21 (107.53, 231.53) | Z=-1.33 | 0.184 |
| LDH (U/L) | 231.00 (184.00, 322.25) | 235.00 (191.00, 323.00) | 221.00 (178.00, 305.50) | Z=-1.16 | 0.248 |
| ALP (U/L) | 159.65 (92.00, 297.60) | 154.90 (114.60, 242.00) | 164.40 (85.45, 308.60) | Z=-0.59 | 0.555 |
| Albumin (g/L) | 39.90 (35.98, 41.62) | 39.50 (35.80, 40.90) | 40.40 (36.15, 41.90) | Z=-1.31 | 0.190 |
| CRP (mg/L) | 7.85 (3.40, 26.83) | 10.00 (3.90, 30.30) | 6.70 (3.25, 23.70) | Z=-1.15 | 0.251 |
| Ca^2+^ (mmol/L) | 2.30 (2.19, 2.39) | 2.30 (2.20, 2.39) | 2.30 (2.15, 2.38) | Z=-0.80 | 0.423 |

χ²: Chi-square test, Z: Mann-Whitney test, -: Fisher exact, M: Median, Q₁: 1st Quartile, Q₃: 3st Quartile, NLR: neutrophil-to-lymphocyte ratio, PLR: platelet-to-lymphocyte ratio, LMR: lymphocyte-to-monocyte ratio
